# Efficient 2-Nitrophenol Chemical Sensor Development Based on Ce_2_O_3_ Nanoparticles Decorated CNT Nanocomposites for Environmental Safety

**DOI:** 10.1371/journal.pone.0166265

**Published:** 2016-12-14

**Authors:** Mohammad M. Hussain, Mohammed M. Rahman, Abdullah M. Asiri

**Affiliations:** 1 Chemistry Department, King Abdulaziz University, Faculty of Science, Jeddah, Saudi Arabia; 2 Center of Excellence for Advanced Material Research (CEAMR), King Abdulaziz University, Jeddah, Saudi Arabia; Institute of Materials Science, GERMANY

## Abstract

Ce_2_O_3_ nanoparticle decorated CNT nanocomposites (Ce_2_O_3_.CNT NCs) were prepared by a wet-chemical method in basic medium. The Ce_2_O_3_.CNT NCs were examined using FTIR, UV/Vis, Field-Emission Scanning Electron Microscopy (FESEM), X-ray electron dispersive spectroscopy (XEDS), X-ray photoelectron spectroscopy (XPS), and powder X-ray diffraction (XRD). A selective 2-nitrophenol (2-NP) sensor was developed by fabricating a thin-layer of NCs onto a flat glassy carbon electrode (GCE, surface area = 0.0316 cm^2^). Higher sensitivity including linear dynamic range (LDR), long-term stability, and enhanced electrochemical performances towards 2-NP were achieved by a reliable current-voltage (I-V) method. The calibration curve was found linear (*R*^*2*^ = 0.9030) over a wide range of 2-NP concentration (100 pM ~ 100.0 mM). Limit of detection (LOD) and sensor sensitivity were calculated based on noise to signal ratio (~3N/S) as 60 ± 0.02 pM and 1.6×10^−3^ μAμM^-1^cm^-2^ respectively. The Ce_2_O_3_.CNT NCs synthesized by a wet-chemical process is an excellent way of establishing nanomaterial decorated carbon materials for chemical sensor development in favor of detecting hazardous compounds in health-care and environmental fields at broad-scales. Finally, the efficiency of the proposed chemical sensors can be applied and utilized in effectively for the selective detection of toxic 2-NP component in environmental real samples with acceptable and reasonable results.

## 1. Introduction

The importance of safety (Environment and health) is a great concern of using semiconductor materials for the detection of toxic chemicals through a well-organized technique. Nanostructure materials are very much efficient and sensitive due to having exceptional properties such as large and active surface area, and spherical size toward volume ratio in comparison with traditional materials in a micro to nano ranges. Generally metal-oxide nanostructures has been attracted great concentration due to their excellent criteria such as higher dynamic surface region, permeability, high porosity, easy fabrication, quantum confinement effect, and stability [1, 2]. Metal oxide conjugated carbon material composites based sensors are broadly using for the detection of poisonous pollutants, in process control of chemicals, and monitoring of air or water pollution in the environment. Removing of toxic compounds from industrial waste water is one of the most important issues in the environmental and health science. Different methods have been developed for the removing of carcinogenic chemicals from industrial waste water effluent. But some issues are still remained unsolved such as preparation of the green NCs at a facile, inexpensive, removing of hazardous compounds in efficiently, and reusability of the stable NCs. In addition, the mesoporous characteristics of the NCs substance allow its superficial recycle without major failure of sensor potentiality and effectiveness. Based on the outstanding adsorption or absorption capability of hybrid NCs and additional recompenses (easy separation, environmentally friendly composition, and reusability), it was already designed a suitable sensor for removing of target toxins from the environmental and industrial wastes. The investigation of phenols and phenolic derivatives in normal water, and effluents is a major significance intended for environmental management, and safety owing toward their existence or emergence from a broad range of human performances. These phenolic derivatives or compound having toxic effect on animals, humans, plants, and they provide an unwanted taste, and odor to consumption water, even at very low concentration. Here, 2-NP is an organic compound under nitro-aromatic group and is widely used in the production of chemical intermediates, explosives, fungicides, gas, herbicides, insecticides, pharmaceuticals, pesticides, petroleum, pigments, rubber chemicals, synthetic dyes, textile and wood [3, 4]. The aromatic nitro-compounds are the toxic substances as well as the detoxification of contaminated water with nitro-aromatic molecules. 2-NP is the most persistent and hazardous organic pollutant of industrial wastewater and exhibits high toxicity or mutagenicity directly or through of its catabolic metabolites for living organisms [5, 6]. Based on conducting polymers, composites and semiconductor oxides, development of selective and efficient chemical sensor is an important issue for the detection and quantification of toxic chemicals and materials [7, 8]. Although traditional techniques have the advantages of sensitivity and accuracy, but most of them suffer difficulties with sample preparation or necessity of molecules derivatization, which limit their utility. Besides that, the electrochemical approach has been attempted for 2-NP determination because of low-cost, simple operation, fast response, sensitive and economical. Nanomaterials had been used in catalytic cracking of naphtha in order to increase the yield of ethylene and propylene [9–10], oxidation of CO [11], antibacterial activity study [12], improvement of electro-catalytic activity and stability of PbO_2_ electrode [13], hydrogen production [14], counter electrode for dye-sensitized solar cells [15], catalyst [16], CO conversion [17], degradation of phenol [18], photocatalytic activity [19], synthesis of spherical YAG [20], catalytic reduction of NO [21], removing of CO [22], catalytic wet-oxidation of 2,4-dichlorophenol solutions [23], enhancement of quantum yield [24], interrelated functionalities of hierarchically nanostructured layers [25], oxidation of methane [26], conversion of a dimensionally mixed ternary NCs [27], waste water treatments [28], and various applications [29–34]. Till to date, various nanostructure or composite materials based electrochemical chemical sensors have been established for the detection of hazardous phenolic compounds. The aim of this study was to synthesize Ce_2_O_3_.CNT NCs by a facile wet-chemical process and fabrication with conducting coating agent towards the detection of 2-NP using dependable I-V technique. It was recognized that the Ce_2_O_3_.CNT NCs fabricated electrode is an efficient and unique approach for the detection of 2-NP using I-V method with short response time in ultrasonically.

## 2. Experimental Section

### 2.1 Materials and Methods

Analytical grade of cerium (III) sulfate, sodium hydroxide (NaOH), disodium phosphate (Na_2_HPO_4_), monosodium phosphate (NaH_2_PO_4_), nafion (5% ethanolic solution), 2-nitrophenol (2-NP), 3-methoxyphenol (3-MP), 4-aminophenol (4-AP), 4-methoxyphenol (4-MP), acetone (Ac), bisphenol A (Bis A), ethanol (EtOH), hydrazine (Hy), melamine (Mel), methanol (MeOH), ammonium hydroxide (NH_4_OH), carbon nanotube (CNT) were purchased from Sigma-Aldrich and used without further purification. FT-IR spectra of the dried Ce_2_O_3_.CNT NCs were performed on a Thermo scientific NICOLET iS50 FT-IR spectrometer (Madison, USA). UV/Vis studies were characterized using evolution 300 UV/Visible spectrophotometer (Thermo scientific). The XPS experiment was conducted on K-α spectrometer (Thermo scientific, K-α 1066) with A1Kα radiation as an excitation resource (Spot-size of beam = 300.0 μm, pressure ~ 10^−8^ Torr, pass energy = 200.0 eV) for the evaluation of binding energy (KeV) of Ce, O, and C. The arrangement, structure, morphology, and elemental size of Ce_2_O_3_.CNT NCs were also investigated by FESEM (JEOL, JSM-7600F, Japan). XRD was conducted to analyze the crystalline pattern of Ce_2_O_3_.CNT NCs under ambient conditions. I-V technique was executed in order to detect 2-NP with fabricated Ce_2_O_3_.CNT NCs by Keithley electrometer (6517A, USA) at normal temperature, where two electrodes (working and counter) directly connected with electrometer.

### 2.2 Preparation of nanocomposites from Ce_2_O_3_ nanoparticles and CNT

The wet-chemical is a conventional and solid-state synthesis method, and widely used in the synthesis of undoped or doped nonmaterial. The products (solids) in this process achieved the smaller grains having shorter duration of phase formation at lower temperature. Based on the wet-chemical procedure [35, 36], active reacting agents such as cerium (III) sulphate [Ce_2_(SO_4_)_3_], CNT and NaOH were used in the preparation of Ce_2_O_3_.CNT NCs. Accordingly, Ce_2_(SO_4_)_3_ (0.1 M, 5.7 g) was dissolved in distilled water (100.0 mL) at a erlenmeyer flask (250.0 mL) and CNT (1.0 wt %, 0.25 μg) was then added in constant stirring. The pH of the resultant solution was controlled at over 10.29 by adding NaOH, and kept for continuous stirring at 90.0°C. After continuous stirring (6 h), the flask washed thoroughly with water and acetone consequently, and then kept for exposure to air (26 h) at room temperature. The resultant greenish product (Ce_2_O_3_.CNT NCs) was dried in the oven at 60.0°C for 24 h, grinding into powders, again dried at 60.0°C in the oven (24 h), and then used for characterizations such as elemental, morphological, optical and structural property, and applied for chemical sensing using I-V technique. The cerium oxide nanoparticles (Ce_2_O_3_ NPs) without CNT were also prepared using the similar procedure under identical conditions. The probable reaction mechanisms for the formation of Ce_2_O_3_.CNT NCs are shown in the reaction (i) to (iv).

$$\begin{array}{l} NaOH_{\left(aq\right)}\to Na^{+}{}_{\left(aq\right)}+OH^{-}{}_{\left(aq\right)}\;(i)\\ Ce_{2}{\left(SO_{4}\right)}_{3}\to 2Ce^{3+}{}_{\left(aq\right)}+3SO_{4}{{}^{2-}}_{\left(aq\right)}\;(ii)\\ 6Na^{+}{}_{\left(aq\right)}+6OH^{-}{}_{\left(aq\right)}+2Ce^{3+}{}_{\left(aq\right)}+3SO_{4}{{}^{2-}}_{\left(aq\right)}\to 2Ce{\left(OH\right)}_{3}{}_{\left(aq\right)}+3Na_{2}SO_{4}{}_{\left(s\right)}↓\;(iii)\\ 2Ce{\left(OH\right)}_{3}{}_{\left(aq\right)}+CNT(dispersed)\to Ce_{2}O_{3}.CNT(s)↓+3H_{2}O_{\left(aq\right)}\;(iv) \end{array}$$

**Fig 1:**
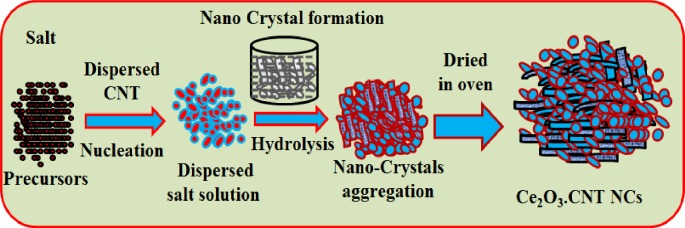
Growth mechanism of Ce_2_O_3_.CNT NCs.

According to the Ce_2_O_3_.CNT NCs growth mechanism, initially nucleus growth takes place by itself and mutual-aggregation, nano-crystal re-aggregated and formed aggregated Ce_2_O_3_ nanocrystal using Ostwald-Ripening method. Nano crystals crystallized and re-aggregated with each other counter parts through Vander-Waals forces in presence of dispersed CNT and Ce_2_O_3_.CNT NCs porous morphology had been reformed (**Fig 1**).

### 2.3 Fabrication of glassy carbon electrode with Ce_2_O_3._CNT NCs

Phosphate buffer, PB (0.1 M, pH = 7) was prepared with addition of Na_2_HPO_4_ (0.2 M) and NaH_2_PO_4_ (0.2 M) in distilled water (200.0 mL). The GCE was fabricated with Ce_2_O_3_.CNT NCs using EtOH and conducting binding agent, nafion. After that it was kept for 3 h until completely dried with uniform thin film formation at room temperature. The fabricated NCs/GCE and platinum wire (Pt) were used as a working and counter electrode respectively.

## 3. Results and Discussion

### 3.1. Choice of nanocomposite materials

Ce_2_O_3_ nanoparticles decorated CNT nanocomposites have employed a great deal of consideration due to their chemical, physical, and optical properties in terms of large-active surface area, high-stability, high porosity, and permeability (porous-tubers nature of CNT), which directly dependent on the structural morphology prepared by uni-molar ratio by reactant precursors [Ce_2_(SO_4_)_3_ and CNT] for making Ce_2_O_3_.CNT NCs in alkaline phase. The Ce_2_O_3_.CNT NCs were synthesized by a wet-chemical method using NaOH as a reducing agent. This technique has several advantages including facile preparation, accurate control the reactant temperature, easy to handle, one-step reaction, and high-porosity as well as porous tuber natures both Ce_2_O_3_ and CNT materials. Optical, morphological, electrical, and chemical properties of Ce_2_O_3_.CNT NCs materials are of huge significant from the scientific aspect, compared to other un-doped materials. Non-stoichiometry, mostly oxygen vacancies, makes it conducting nature in the nanocomposites. The formation energy of oxygen vacancies and metal interstitials in semiconductor is very low and thus these defects form eagerly, resulting in the experimental elevated conductivity of Ce_2_O_3_.CNT NCs compared to other un-doped materials. Ce_2_O_3_.CNT NCs materials have also attracted considerable interest owing to their potential applications in fabricating optoelectronics, electro-analytical, selective detection of bioassays, biological devices, hybrid-composites, electron-field emission sources for emission exhibits, biochemical detections, and surface-enhanced Raman properties etc. Further adsorption and incorporation of cerium oxide nanoparticles into the porous carbon nanotube material offers improved performance due to the increase of conductivity and active surface area of the Ce_2_O_3_.CNT NCs.

### 3.2. Evaluation of optical and structural properties

**Fig 2:**
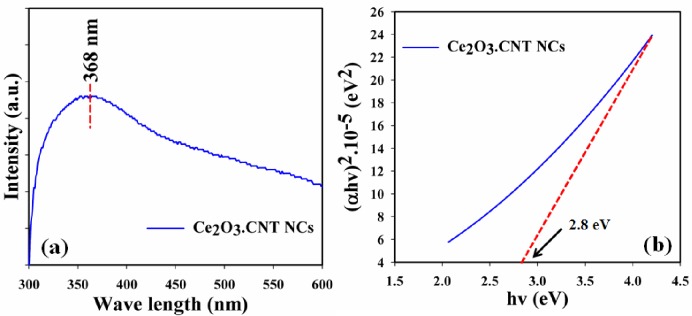
(a-b) UV/Vis. spectrum and regarding band gap energy plot of Ce_2_O_3_.CNT NCs.

The optical characteristic is one of the significant criteria of the assessment of the photo-catalytic activity of the greenish-grown Ce_2_O_3_.CNT NCs. In UV/Vis. phenomena, the external electrons of the atom can absorb radiant energy undergoing transition to higher energy level. The spectrum can be obtained due to the optical absorption of radiant energy in order to achieve band-gap energy of the metal oxide. The UV/Vis. absorption band of the Ce_2_O_3_.CNT NCs was measured in the range of 200 ~ 800 nm, and found a wide absorption band at around 368.0 nm (Fig 2A). Based on the maximum level band absorption, the band-gap energy of the Ce_2_O_3_.CNT NCs was calculated using Eq (v), and according to the Tauc’s equation [direct band-gap rule, (vi)], (*αhv*)^2^ *vs hv* were plotted and then extrapolated to the x-axis. From the extrapolated curve, the band-gap energy of the Ce_2_O_3_.CNT NCs was found as ~ 2.8 eV (Fig 2B). Where, E_bg_: Band-gap energy, λ_max_: Maximum absorption wavelength, α: Absorption coefficient, A: Constant related to the effective mass of the electrons, r: 0.5 (Direct transition), h: Plank’s constant, v: Frequency [37].

$$E_{bg}=1240/\lambda_{max}\;\left(eV\right)$$ (v)

$${\left(\alpha hv\right)}^{1/r}=A\;\left(hv\text{-}E_{bg}\right)$$ (vi)

**Fig 3:**
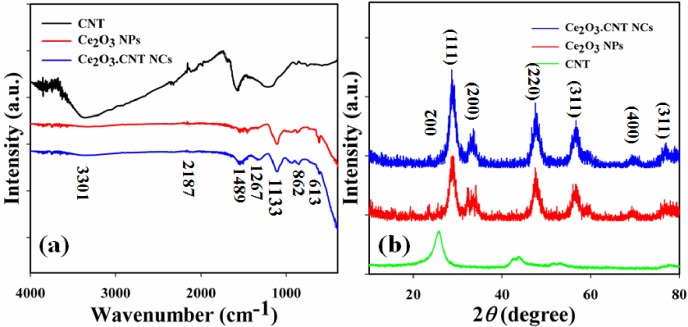
(a) FTIR spectra and (b) XRD pattern of the CNT, Ce_2_O_3_ NPs and Ce_2_O_3_.CNT NCs.

The greenish grown Ce_2_O_3_.CNT NCs were characterized in points of atomic and molecular vibration. In order to identify the functional properties, FTIR spectra were recorded under normal condition in the region of 400 ~ 4000 cm^-1^. The FT-IR spectrum of the Ce_2_O_3_.CNT NCs (Fig 3A) shows peaks at 3301 (br), 2187 (w), 1489 (w), 1267 (m), 1133 (m), 862 (w), and 613 (w) cm^-1^ which indicated the presence of O−H, −C≡C−, C = O, C−H, ˃C = C˂, C−H, −Ce = O respectively in the NCs. The observed peak at 613 cm^-1^ demonstrated the formation of metal-oxide bond (−Ce = O) which denoted the configuration of the Ce_2_O_3_.CNT NCs [38].

The XRD spectrum was recorded to study the crystalline and crystal properties of the synthesized Ce_2_O_3_.CNT NCs. All the peaks found in the spectrum were assigned by using the JCPDS file (34–0394) that was a pure cubic phase (*Fm3m*). The observed characteristics peaks with denoted for 2*θ* values at 22.5 (002), 28.5 (111), 32 (200), 48 (220), 57 (311), 70 (400) and 78 (331) degrees (Fig 3B). The found Ce_2_O_3_ lattice constant, 5.418 Å was almost same with theoretical value 5.411 Å. Here peak at 002 is denoted for CNT. So, these parameters indicated that a significant quantity of crystalline Ce_2_O_3_ with CNT is presented in the nanocomposites [39, 40].

### 3.3 Characterization of morphological and elemental properties

**Fig 4:**
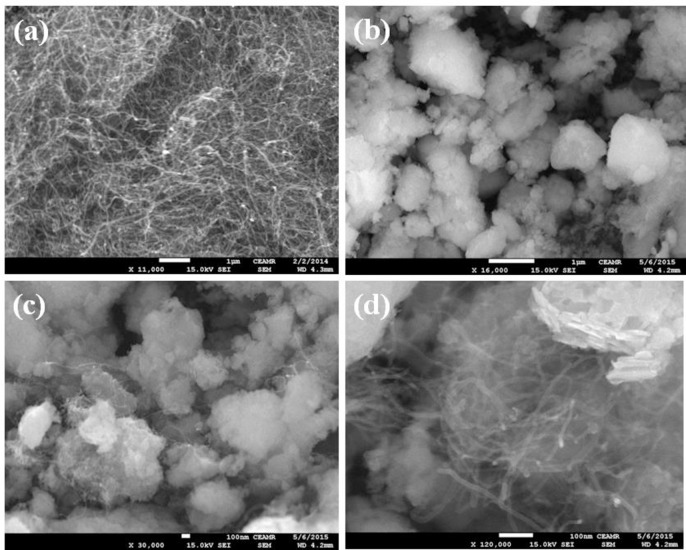
Magnified FESEM images, (a) CNT, (b) Ce_2_O_3_ NPs, and (c-d) Ce_2_O_3_.CNT NCs.

FESEM is one of the excellent techniques to characterize the morphology of nanocomposite and nanostructure compounds. The elemental and morphological properties of the black CNT and greenish grown Ce_2_O_3_ NPs and Ce_2_O_3_.CNT NCs were examined using FESEM equipped with XEDS respectively. The typical shape of black CNT, Ce_2_O_3_ NPs and greenish grown Ce_2_O_3_.CNT NCs at low to high magnified images were recorded using FESEM (Fig 4A–4D). The magnified images indicated that Ce_2_O_3_ was aggregated with a bright contrast and well dispersed on the surface of CNT (Fig 4C and 4D). The conductance of CNT could be increased with the incorporation of Ce_2_O_3_, which correlated the calculation of E_bg_ of two samples.

**Fig 5:**
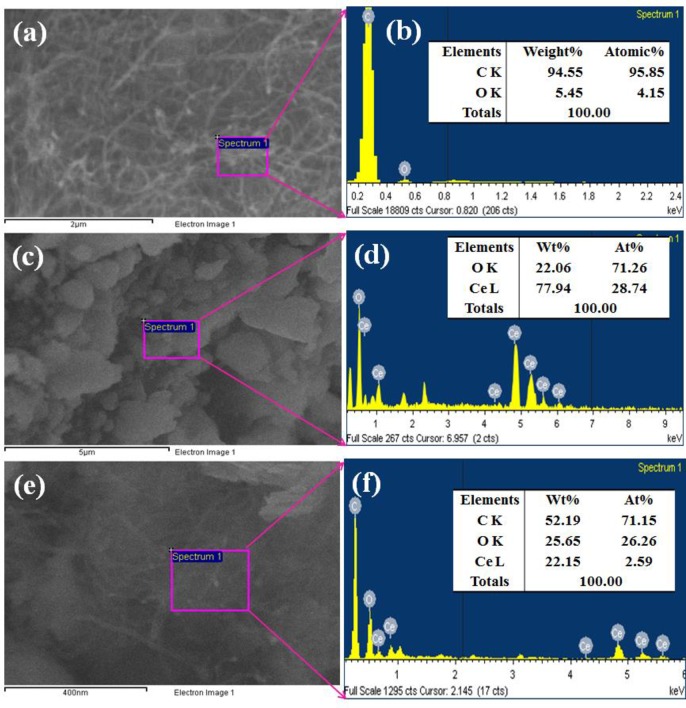
(a-f) Elemental composition of CNT, Ce_2_O_3_ NPs and Ce_2_O_3_.CNT NCs.

According to the XEDS analysis, carbon (C), oxygen (O) and cerium (Ce) were present in the synthesized greenish grown Ce_2_O_3_ NPs and Ce_2_O_3_.CNT NCs (Fig 5A–5C). It was clearly revealed that the prepared NPs contains O (22.06), Ce (77.94) and NCs consist of C (52.19), O (25.65) and Ce (22.15) wt% respectively (Fig 5B–5D). Based on the elemental analysis, carbon was absent in NPs but present in the NCs that meant CNT was properly dispersed with bright contrast on Ce_2_O_3_ NPs. There was no additional peaks found associated with impurities in the FESEM attached with XEDS, which indicated that the NCs composed of C, O and Ce only. A comparison in weight (%) among CNT, Ce_2_O_3_ NPs, and Ce_2_O_3_.CNT NCs is given in Table 1.

**Table 1:** Comparison of weight, and binding energies among materials.

Materials	Weight (%)			Binding energies (eV)		
	C	O	Ce	C1s	O1s	Ce 3d_5/2_
CNT	94.55	5.45	-	285.0	-	-
Ce_2_O_3_ NPs	-	22.6	77.94	-	537.0	888.0
Ce_2_O_3_.CNT NCs	52.19	25.65	22.15	290.0	532.0	883.0

### 3.4. Examination of binding energy

**Fig 6:**
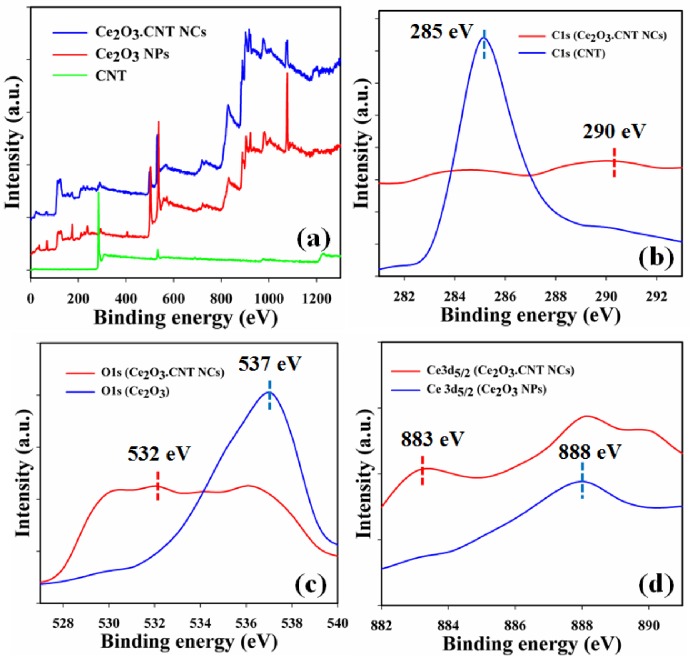
XPS spectra: (a) Ce_2_O_3_.CNT NCs, (b) C1s, (c) O1s, and (d) Ce3d_5/2_ level achieved with Kα1 radiation.

XPS is a significant spectroscopic method for quantitative evaluation which indicated that the presence the chemical nature of the element within NCs. XPS spectrum can be achieved by irradiating materials under X-ray beam and concurrently measures the kinetic energy as well as electrons number of the sample [1]. According to the XPS spectra, carbon, oxygen and cerium were present in the prepared NCs (Fig 6A). The C1s spectrum recognized the main peak at 290.0 eV for carbon (Fig 6B). A major peak at 532.0 and 883.0 eV were found for lattice oxygen (O1s) and spin orbit cerium (Ce 3d_5/2_), which denoted that oxygen (O^2-^) and cerium (Ce^3+^) were present in the Ce_2_O_3_.CNT NCs (Fig 6C and 6D). A comparison of the binding energies among C, O, and Ce in the CNT, NPs and NCs are presented in Table 1.

## 4. Applications

### 4.1. Detection of 2-NP using Ce_2_O_3_.CNT nanocomposites

**Fig 7:**
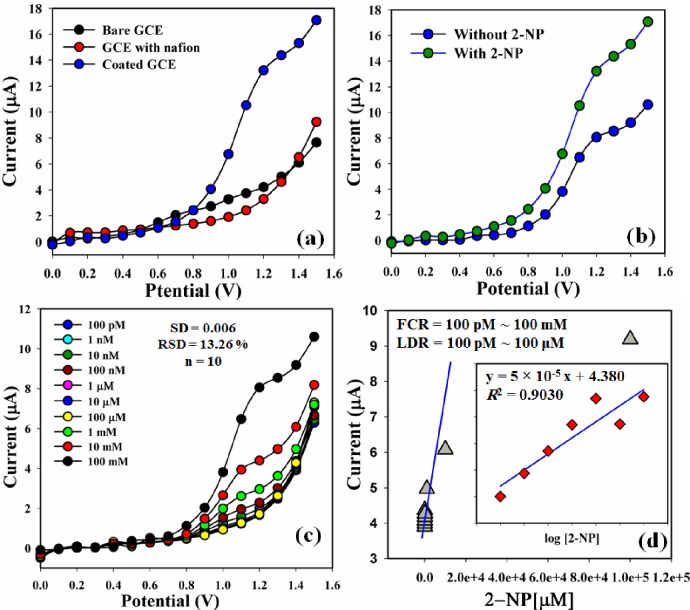
I-V responses of Ce_2_O_3_.CNT NCs/GCE: (a) Bare, GCE with nafion and coated electrode, (b) Absence and presence of 2-NP, (c) Concentration variation, and (d) Calibration curve, [inset: Linear dynamic range curve].

The vital application of Ce_2_O_3_.CNT NCs assembled onto an electrode as a chemical sensor has been explored for the identification of chemicals that are environmentally toxic. The NCs materials have been reported earlier as chemical sensors [41–42]. The Ce_2_O_3_.CNT NCs sensors have many advantages such as chemically stable, consistent in air, nontoxic, large surface area, biologically safe and simple to assemble. The current response of the Ce_2_O_3_.CNT NCs considerably changed during adsorption 2-NP as target analyte in the I-V technique. On the basis of potential range (0.0 ~ 1.5 V), the current responses for the uncoated bare GCE, and coated with Ce_2_O_3_.CNT NCs on the working electrode surface are shown in Fig 7A. In comparison, the current signal was much affected with coated GCE which indicated that the differences of the current responses between bare and coated GCE (Fig 7A). The changes of current without 2-NP (blue-dotted) and with 2-NP (green-dotted) of the Ce_2_O_3_.CNT NCs modified electrode are shown in Fig 7B. A significant enhancement of current was achieved with 2-NP owing to the existence of NCs which give a high surface area, better absorption and adsorption capacity onto the porous NCs surfaces. The responses of the Ce_2_O_3_.CNT NCs modified electrode were examined with the different concentration of 2-NP (100 pM ~ 100 mM) which shown the changes of current of the fabricated electrode as a function of 2-NP concentration under normal condition (SD = 0.006, RSD = 13.26%, and n = 10). It was observed that the current responses enhanced regularly from lower to higher concentration of the target analyte (Fig 7C). A good range of the analyte concentrations were examined from the lower to higher potential (0.0 ~ 1.5 V) to observe of the possible analytical limit. The calibration curve at 1.4 V was plotted from the final concentration range (100 pM ~ 100 mM) of 2-NP. Regression co-efficient (*R*^*2*^ = 0.9030), LOD (60 ± 0.02 pM) and sensitivity (~1.6 ×10^−3^ μAμM^-1^cm^-2^) at signal to noise ratio = 3, and LDR (100.0 pM ~ 100.0 μM) were calculated from the calibration curve (Fig 7D).

Due to the important characteristics of the NCs material, the resistance value of the Ce_**2**_O_**3**_.CNT NCs modified GCE chemical sensor can be decreased with enhancing of active surface area [43, 44]. Actuality, adsorption of oxygen (O_**2**_) displayed a significant liability in the electrical quality of the Ce_**2**_O_**3**_.CNT NCs. Adsorption of oxygen ion (O_**2**_^**-**^) eradicated the conduction of electrons, and enhanced the resistance of Ce_**2**_O_**3**_.CNT NCs. Active oxygen species (O_**2**_^**−**^, and O^**−**^) may be adsorbed onto the surfaces of NCs at normal condition, and the amount of such chemically adsorbed oxygen species strongly depended on the porous property. At normal condition, O_**2**_^**−**^ is chemically adsorbed, while in NCs morphology, oxygen species (O_**2**_^**−**^, and O^**−**^) that are chemically adsorbed vanishes rapidly [45, 46]. 2-NP sensing method of Ce_**2**_O_**3**_.CNT NCs chemical sensor based on doped Ce_**2**_O_**3**_, that is shown due to the redox reaction of the NCs. Based on the dissolved oxygen in electrolyte solution or air surface of the neighbouring environment, the reactions [(vii)—(ix)] may be consummated as follows.

$$2e^{\text{-}}\;\left(Ce_{2}O_{3}.CNT\;NCs\right)+O_{2}\to O_{2}{}^{2\text{-}}$$ (vii)

$$2e^{\text{-}}\;\left(Ce_{2}O_{3}.CNT\;NCs\right)+O_{2}{}^{2\text{-}}\to 2O^{2\text{-}}$$ (viii)

$$2O^{2\text{-}}↔O_{2}+2e^{\text{-}}$$ (ix)

**Fig 8:**
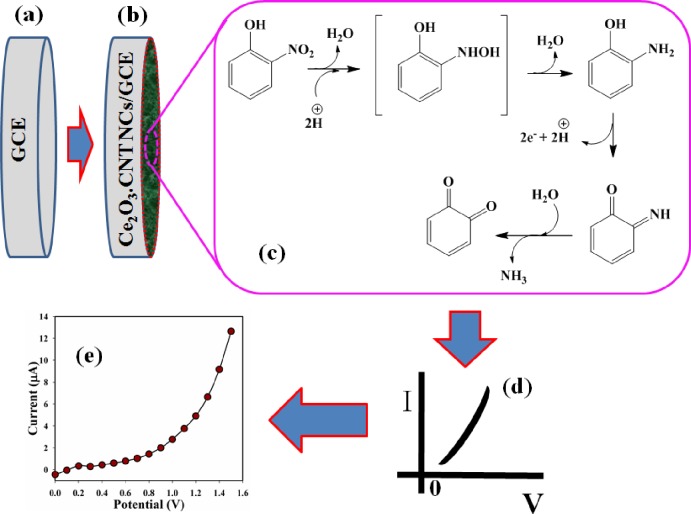
(a) Bare GCE, (b) Fabricated GCE with NCs and Nafions, (c) Possible mechanism of 2-NP, (d) Expected I-V curve, and (e) Observed I-V responses.

The above reactions were generalized in the electrolyte system or air/liquid interface or nearer environment due to the tiny carrier concentration that increased the resistance. 2-NP sensitivity towards Ce_2_O_3_.CNT NCs can be recognized to the higher oxygen lacking conducts to increase the oxygen adsorption. More oxygen adsorbed on the Ce_2_O_3_ doped NCs sensor surface, more oxidizing potentiality, and faster oxidation of 2-NP can be occurred. The action of 2-NP can be extremely immense as compare to other toxic chemicals with the surface under identical conditions [47, 48]. 2-NP can be converted into cyclohexa-3, 5-diene-1, 2-dione under reduction and subsequently oxidation onto the surface of Ce_2_O_3_.CNT NCs whether in exterior or interior of particle-surface or interior-tube. Then subsequent oxidation reaction is held by releasing of free electrons towards the conduction band of Ce_2_O_3_.CNT NCs, which enhanced the current responses against the selective voltages. These free electrons are the main factors to increase the resultant I-V responses in electrochemical approaches (**Fig 8**) [49, 50].

**Fig 9:**
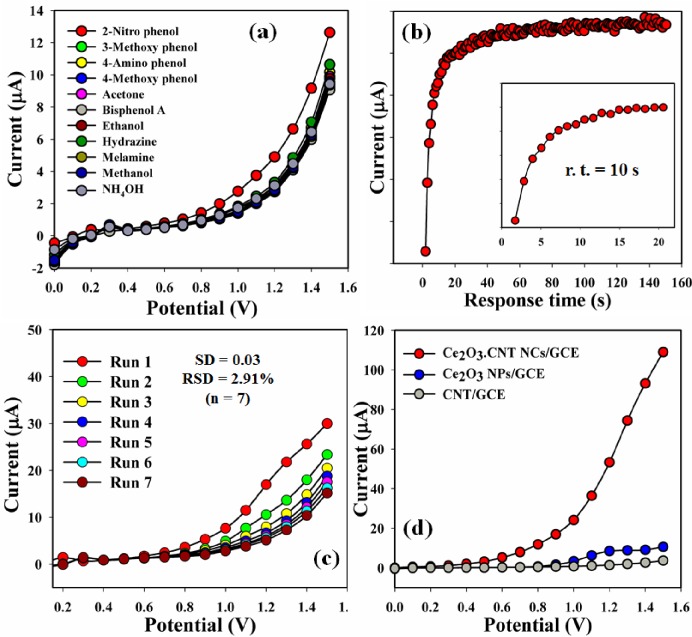
I-V responses of Ce_2_O_3_.CNT NCs coated electrode for 2-NP sensing: (a) Selectivity, (b) Reproducibility study, and (c) Control experiment.

The sensing performances (selectivity) of Ce_2_O_3_.CNT NCs/GCE were performed with different chemicals such as 2-NP, 3-MP, 4-AP, 4-MP, Ac, Bis A, EtOH, Hy, Mel, MeOH, and NH_4_OH, 2-NP shown maximum current responses, and hence it had been noticeably reported that the sensor was more selective toward 2-NP compared with other chemicals (Fig 9A). Response time (R. t. = 10 s) of the Ce_2_O_3_.CNT NCs/GCE sensor towards 2-NP was calculated from the convenient concentration deviation graph (Fig 9B). In order to identify the reproducibility and storage capacity, the I-V responses of the Ce_2_O_3_.CNT NCs coated electrode was performed up to 2 weeks. In this regards, a series of seven consecutive measurements of 2-NP concentration (1.0 μM) were recorded and found good reproducible responses towards the Ce_2_O_3_.CNT NCs/GCE sensor under different conditions [SD = 0.03, RSD = 2.91%, and n = 7 (Fig 9C)]. The fabricated Ce_2_O_3_.CNT NCs electrode substrate washed gently after each experiment, and it was reported that the current responses were not significantly changed. The sensitivity retained almost similar as the preliminary responses up to 2 weeks, and after that the responses of the fabricated electrode decreased steadily. From the above results it is clearly suggested that the fabricated sensor can be used without any significant loss of sensitivity for several weeks. On a further increase the sensitivity of 2-NP analyte concentration onto Ce_2_O_3_.CNT NCs film, which has low-dimensional crystallite size and low lattice disorder of Ce_2_O_3_ onto decorated CNT, presents a more rapid increase the response due to much larger surface covered by analyte onto the NCs/GCE sensor surface. Due to high specific surface area, the Ce_2_O_3_.CNT NCs provide a favorable micro-environment for the 2-NP analyte detection with good quantity [51,52]. The high sensitivity of NCs/Nafion/GCE provides high electron communication features which enhanced the direct electron transfer between the active sites of Ce_2_O_3_ conjugated CNT NCs onto GCE. The high sensitivity of the fabricated NCs/Nafion/GCE can be attributed to the excellent absorption (porous surfaces in NCs/Nafion/GCE) and adsorption ability, high catalytic-decomposition activity, and good biocompatibility of the NCs. For these reasons, the estimated sensitivity of the fabricated sensor is relatively higher and detection limit is comparatively lower than previously reported 2-NP sensors based on other nano-composite or nano-materials modified electrodes. Control experimentation was conducted using CNT/GCE, Ce_2_O_3_/GCE, and Ce_2_O_3_.CNT NCs/GCE with 2-NP concentration (100 nM) and a considerable increase of current response observed for the Ce_2_O_3_.CNT NCs/GCE compared with Ce_2_O_3_/GCE, and CNT/GCE (Fig 9D). A comparison of the sensor performances for 2-NP detection using different modified electrodes by electrochemical approach is presented in Table 2 [53–61].

**Table 2:** Sensor performances towards 2-NP detection using different electrochemical approaches.

Materials	Methods	LDR (pM ~ μM)	DL (nM)	Sensitivity (μA μM^-1^cm^-2^)	Linearity (*R*^*2*^)	Ref.
Mn-ZnS QDs	CL	0.1 ~ 40 μM	76.0	-	-	[53]
Ag2O NPs/AuE	I-V	1.0 μM ~ 0.5 mM	0.19 μM	0.0474	0.9873	[54]
CuO Nanohybrides	I-V	1.0 nM ~ 1.0 mM	0.67	0.045	0.7941	[55]
Poly(safranine) Film Electrode	CV/LSV	8.0× 10^‒8^ ~ 4.0×10^‒5^ M	3.0×10^‒8^ M	-	0.9880	[56]
Spinel ZnMn_2_O_4_	I-V	50.0 μM ~ 0.05 M	20.0 μM	1.5	0.7599	[57]
Immunoassay	FS	5, 1000 μg/L	3.5	5.7 mg/L	-	[58]
GO sensors	CV	0.1 ~ 120 μM	0.02 μM	-	-	[59]
B-Diamond Electrodes	SWV	-	8.4 mM	0.3943	0.9991	[60]
Mn_2_O_3_-ZnO NPs/AgE	I-V	100 ~ 50.0	~0.83	~0.6667	0.9773	[61]
Ce_2_O_3_.CNT NCs	I-V	100 ~ 100	60 ± 0.02 pM	1.6 ×10^−3^	0.9030	This work

CL: Chemiluminescence, FS: Fluorescence Spectroscopy.

### 4.2. Real sample analysis

Regarding confirmation of the validity of I-V method, the Ce_2_O_3_.CNT NCs/GCE had been used to find out the 2-NP in various real samples. In real sample study, a standard addition method was used to estimate the concentration of 2-NP in water samples that were collected from different sources. A fixed amount (~25.0 μL) of each real sample was mixed and analyzed in PB (10.0 mL) using fabricated Ce_2_O_3_.CNT NCs/GCE. The found results regarding 2-NP detection are presented in Table 3, and apparently confirmed that the proposed Ce_2_O_3_.CNT NCs/GCE approach is satisfactory, reliable, and suitable for analyzing real samples using I-V system.

**Table 3:** Determination of 2-NP concentration at different real samples using modified Ce_2_O_3_.CNT NCs/GCE.

Real samples	Observed current (μA)				Conc. (μM)	SD (n = 3)
	R1	R2	R2	Average		
Industrial effluent	18.58	15.72	14.83	16.38	3.84	1.96
PC baby bottle	20.09	15.39	13.75	16.41	3.84	3.29
PC bottle safa	13.78	10.42	9.58	11.26	2.64	2.22
PVC food packaging bag	17.95	20.23	16.92	18.37	4.30	1.69
Red sea water	18.74	15.39	14.51	16.21	3.80	2.23
Tape water	14.41	11.00	10.00	11.80	2.76	2.31

R: Reading, SD: Standard deviation.

## 5. Conclusion

Ce_2_O_3_.CNT NCs were prepared using active reducing agents by a wet-chemical process, which was simple, efficient, reliable, and economical. The elemental, morphological, optical, and structural properties were examined using various conventional methods, such as FTIR, XEDS, XRD, FESEM, XPS, and UV/visible spectroscopy. Ce_2_O_3_.CNT NCs electrode was fabricated by an easy fabrication method, which exhibited higher sensitivity towards 2-NP. A selective and sensitive 2-NP sensor based on electrode embedded with Ce_2_O_3_.CNT NCs having conducting coating binder was prepared in successfully. The analytical parameters of the fabricated 2-NP sensor were excellent in terms of LOD, LDR, sensitivity, and short response time. The Ce_2_O_3_.CNT NCs electrode reflects higher sensitivity (~1.6×10^−3^ μAμM^-1^cm^-2^) and lower detection limit (60 ± 0.02 pM) using a reliable I-V method. Ce_2_O_3_.CNT NCs sensor was finally tested with few real samples and obtained satisfactory results. A well-organized technique can be introduced from this novel approach for the development of efficient chemical sensor for the detection of hazardous materials in the environmental and health care arena at a wide range.
